# *Neurog3* misexpression unravels mouse pancreatic ductal cell plasticity

**DOI:** 10.1371/journal.pone.0201536

**Published:** 2018-08-09

**Authors:** Andhira Vieira, Bastien Vergoni, Monica Courtney, Noémie Druelle, Elisabet Gjernes, Biljana Hadzic, Fabio Avolio, Tiziana Napolitano, Sergi Navarro Sanz, Ahmed Mansouri, Patrick Collombat

**Affiliations:** 1 Univ. Nice Sophia Antipolis, Inserm, CNRS, iBV, Nice, France; 2 Max-Planck Institute for Biophysical Chemistry, Department of Molecular Cell Biology, Am Fassberg, Göttingen, Germany; 3 Department of Clinical Neurophysiology, University of Göttingen, Göttingen, Germany; Vrije Universiteit Brussel, BELGIUM

## Abstract

In the context of type 1 diabetes research and the development of insulin-producing β-cell replacement strategies, whether pancreatic ductal cells retain their developmental capability to adopt an endocrine cell identity remains debated, most likely due to the diversity of models employed to induce pancreatic regeneration. In this work, rather than injuring the pancreas, we developed a mouse model allowing the inducible misexpression of the proendocrine gene *Neurog3* in ductal cells *in vivo*. These animals developed a progressive islet hypertrophy attributed to a proportional increase in all endocrine cell populations. Lineage tracing experiments indicated a continuous neo-generation of endocrine cells exhibiting a ductal ontogeny. Interestingly, the resulting supplementary β-like cells were found to be functional. Based on these findings, we suggest that ductal cells could represent a renewable source of new β-like cells and that strategies aiming at controlling the expression of *Neurog3*, or of its molecular targets/co-factors, may pave new avenues for the improved treatments of diabetes.

## Introduction

Type 1 diabetes mellitus (T1DM) results from the auto-immune-mediated loss of pancreatic β-cells, such ablation leading to hyperglycemia and severe cardiovascular complications [1–3]. Currently, daily exogenous insulin supplementation represents the main therapy for T1DM patients. Unfortunately, this approach does not allow for a precise regulation of the glycemia and complications often arise with time. In addition to T1DM patients, previous reports demonstrated that 27% of patients with type 2 diabetes (T2DM) eventually require insulin supplementation to maintain their blood glucose levels within normal ranges, a proportion which is likely to increase over time [4]. In this context, finding alternatives to daily injections of insulin has become a major research goal. Towards this aim, understanding the molecular mechanisms underlying β-cell (neo)genesis during development or throughout adulthood could pave the way towards the establishment of β-cell replacement therapies for diabetes. Thus, it was previously demonstrated that, during pancreas morphogenesis, bi-potential pancreatic precursor cells can either give rise to ductal or endocrine cells [5]. Since both tissues share a common ancestor, it has been questioned whether adult ductal cells could adopt an endocrine cell identity. Contradictory studies have been published on this issue [6–22], most likely due to the diversity of models used to induce pancreatic injury/regeneration. While the conclusions were conflicting, it is important to note that a common feature of the studies suggesting duct-to-endocrine conversion, both in embryos and in adults, is the re-expression of the endocrine developmental gene *Neurog3* in ductal cells [6–12]. Therefore, to provide additional insight into the potential of ductal cells to adopt an endocrine cell identity, rather than injuring the pancreas, we developed an animal model allowing the inducible misexpression of *Neurog3* in adult ductal cells and their lineage tracing. The main goal of this work was to establish whether or not pancreatic adult ductal cells retained the developmental capability to give rise to endocrine cells upon the sole ectopic expression of *Neurog3*. Here, we report an islet hypertrophy in mice misexpressing *Neurog3* in ductal cells. Importantly, this hypertrophy is attributed to a progressive increase in α-, β- and δ-cell counts which respect the endogenous endocrine cell ratios when compared to control pancreata. Lineage tracing experiments demonstrate that continuously generated supplementary endocrine cells derive from *Neurog3*-misexpressing ductal cells. These newly-formed endocrine cells are found to be functional and the expression of *bona fide* endocrine markers appears homogenous among the islet cells. Interestingly, the maintained expression of *Neurog3* in mature insulin-producing cells does not impair their function.

## Materials and methods

### Ethics statement

All mouse work was conducted according to French ethical regulations. This project received the approval from the “Ciepal-Azur” local ethics comity (NCE/2011-22).

### Animal procedures

Mice were maintained on a 12-hour light/dark cycle and were provided with standard chow and water *ad libitum*. Animal care and experimental procedures were conducted according to French ethical regulations. Wild-type (WT) 129/sv mice were obtained from Charles River and from Taconic laboratories. *Neurog3*-misexpressing mice (N3OE) were generated in house using the strategy described in **S2A Fig**. This mouse line was crossed with an Insulin-Cre mouse line (**S2C Fig**) [13] and HNF1b-CreER mice [7] (**S2B Fig**). The transgenic mice were genotyped using the following primers: Cre-F *atg ctt ctg tcc gtt tgc cg*; Cre-R *cct gtt ttg cac gtt cac cg*; CAG-F *gca gcc att gcc ttt tat ggt aa*; CAG-R *gat gga gaa ggg gac ggc ggc gc*. HNFN3OE mice were treated with tamoxifen (Sigma) at a concentration of 20mg/ml for 5 consecutive days provided by oral gavage (4mg/day) and then in drinking water at the estimated dose of 250 to 400ng/day for the specified durations (Tamoxifen Citrate; Biogaran® - final concentration 50mg/l). One week of washout was observed between the end of Tamoxifen induction and the sacrifice of the animals. Long-term effect of tamoxifen treatment was assessed over a period of 3 months and revealed no significant difference in islet size, number and composition between untreated wild-type, treated wild-type or negative-transgene littermates and untreated HNFN3OE animals (data not shown). Therefore, the controls in this study were untreated HNFN3OE age-matched mice. Only males were used for the analysis, with a minimum number of n = 3 animals per condition. To assess cellular proliferation, animals were treated with BrdU in drinking water for 10 days prior to examination (1 mg/ml solution).

### Intraperitoneal glucose tolerance test (IPGTT)

Transgenic and control age-matched mice were fasted overnight before intraperitoneal injection with a solution of D-Glucose (Sigma; 2g/kg in H_2_O). Glycemia was measured with a ONETOUCH Vita Glucometer (Life Scan, Inc, CA) before glucose administration and then monitored every 30min until all mice reached euglycemia

### Quantitative RT-PCR (qPCR) analyses

qPCR analyses were performed were performed using the QuantiTect SYBR Green RT-PCR kit (Roche) and Qiagen primers on a LightCycler 480 ® instrument (Roche Life Science) using the housekeeping gene *GAPDH* as internal control for normalization purposes. The qPCR reactions contained 5μL 2x SYBR Green Supermix, 0.5μL Primer Assay, 3μL H_2_O and 1.5μL of cDNA (diluted 1/20 after previous step). The program used for the RT-PCR was the following, with a fluorescence acquisition step at the end of each cycle: 95°C for 10’, 95°C for 10”, 45 cycles of [60°C for 45”, 72°C for 1” and 40°C for 10”] and melting curves at 40°C for 10”.

### Immunohistochemistry

Pancreatic tissue was isolated from euthanized mice and fixed for 30min at 4°C in paraformaldehyde and embedded in paraffin.

The primary antibodies used in this study were as follow: anti-glucagon (mouse, Sigma; rabbit, R&D Systems), anti-insulin (guinea pig, Linco), anti-somatostatin (rat, Chemical International), anti-Neurog3 (mouse, Millipore; guinea pig, kindly provided by M. Sander), anti-BrdU (mouse, Roche), anti-Nkx6.1 (rabbit, NovoNordisk), anti-Pdx1 (rabbit, kindly provided by C. Wright), anti-NeuroD1 (rabbit, Millipore), anti-PC1/3 (rabbit, Millipore), anti-Glut2 (rabbit, Chemical International) and anti-Rfx6 (rabbit, kindly provided by G. Gradwohl). The appropriated secondary antibodies applied were purchased from Molecular Probes and Jackson ImmunoResearch. The images were acquired on a ZEISS AxioImager M2 with motorized plate or a Leica DM5500 TCS SPE. Ultrastructural analyses were performed as previously reported [14].

### β-galactosidase staining

To visualize β-galactosidase activity, we either used X-Gal staining (5-bromo-4-chloro-3-indolyl-β-D-galactopyranoside solution: 625μL of 400mg/mL XGal (Invitrogen), 250μL 500mM K_3_Fe(CN)_6_ (Sigma), 500μL 250mM K_4_Fe(CN)_6_ (Sigma), 100μL 0.5 MgCl_2_, 2.5mL 10x PBS, ddH_2_O qsp 25mL) or Salmon-Gal staining (6-chloro-3-indolyl-β-D-galactopyranoside solution: 1mg/mL Salmon Gal and 3.3μL/mL TetraNitro Blue Tetrazolium Chloride–dissolved in absolute ethanol at 100mg/mL–in phosphate buffer solution). Images were acquired using a LEICA DM 6000B.

### Counting and data analysis

Islet composition were measured *in silico* by counting and measuring objects with the Volocity software. Pancreatic tissue and islet areas were measured using the ZEISS Axiovision software. Briefly, every 10^th^ slide was immunostained against insulin, somatostatin and glucagon. The total area of pancreatic tissue was measured on a mosaic picture and each islet was photographed at higher magnification using the same settings for all animals and genotypes. To determine the size of insulin^+^, somatostatin^+^ and glucagon^+^ cell populations, their areas were measured on individual islets, added together to determine the total area per section and prorated to the corresponding pancreatic section area. Transgenic animals and age-matched controls were then compared to assess the relative variation in each population size, as well as the changes in islets size and number. The proportions of the different endocrine cell populations were assessed by comparing their area within each islet to the global area of said islet. BrdU positive and β-Galactosidase expressing cells were counted manually.

### Data analysis

All values are depicted as mean ± SEM of data from at least three animals. Data were analyzed using GraphPrism 6 software. Normality was tested using a D’Agostino–Pearson omnibus normality test, and appropriate statistical tests were performed (Mann-Whitney or unpaired *t*-tests). Results are considered significant if P < 0.001 (***), P < 0.01 (**), and P < 0.05 (*).

## Results

### Generation and characterization of animals misexpressing *Neurog3* in adult ductal cells

Aiming to ectopically express *Neurog3* in ductal cells in an inducible and conditional fashion, we used the HNF1b-CreER mouse line [7] whose transgene is composed of the *HNF1b* promoter upstream of the tamoxifen- (Tam-) inducible *CreER(T2) recombinase* (**S1A Fig**). The efficiency of *Cre* expression in ductal cells was validated by crossing HNF1b-CreER mice with the ROSA-β-Gal reporter mouse line (harboring a transgene containing the *Rosa26 promoter* upstream of a *loxP-Neomycin resistance-Stop-loxP-β-galactosidase* cassette, **S1A Fig**—[15]). The characterization of the resulting HNF1b-CreER::ROSA-β-Gal double transgenic mice (treated for 7 days with Tam by oral gavage) using a combination of immunohistochemistry (**S1B Fig**) and X-GAL staining (**S1C and S1D Fig**) demonstrated a permanent labeling with the β-galactosidase reporter of an average of 47±18% of ductal cells (**S1E Fig**). These results were in accordance with previous studies which reported a labeling of 40 to 65% of ductal cells [7].

**Fig 1:**
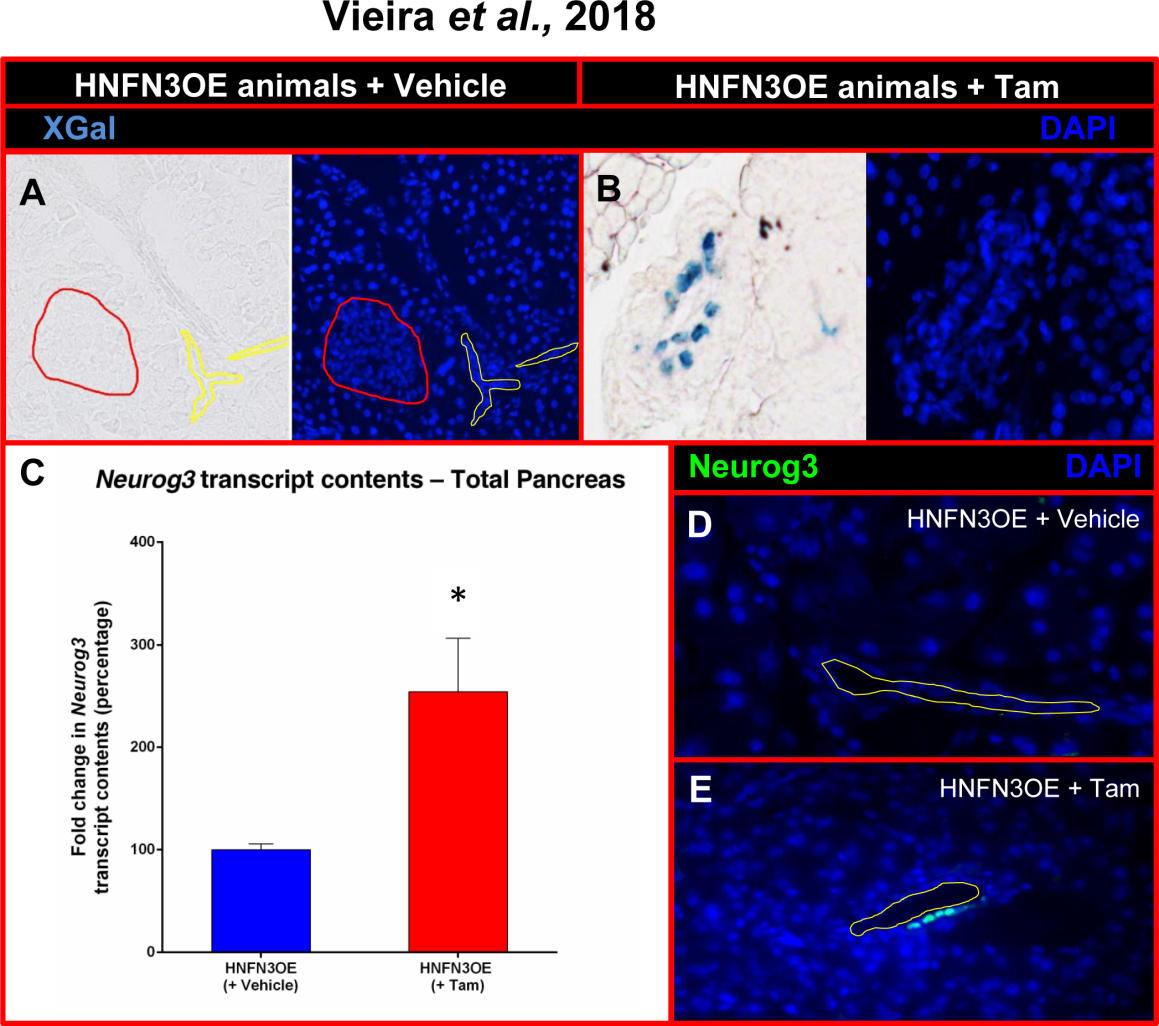
Analysis of the *Neurog3* misexpression efficiency in HNFN3OE mice following short-term tamoxifen induction. (**A-B**) β-galactosidase activity assessment in the pancreata of HNFN3OE animals treated with vehicle (**A**) or Tam (**B**) for 2 weeks. A clear activity is noted solely in ductal cells. (**C**) Quantitative analysis of *Neurog3* transcript levels by qPCR (n = 6 animals for each condition) outlining a 2.5-fold increase in the pancreata of Tam-treated HNFN3OE animals compared to controls. Statistics were performed using the Mann-Whitney test (**D-E**) By means of immunohistochemical analyses using antibodies raised against Neurog3, Neurog3-expressing cells are detected within the ductal epithelium of HNFN3OE animals treated with Tam for only 2 weeks (**E**), whereas Neurog3^+^ cells cannot be detected in their vehicle-treated counterparts (**D**). For clarity, when required, the ductal lumen is outlined with yellow lines and islets with red lines.

We concomitantly generated a conditional *Neurog3*-misexpressing mouse line (N3OE) by pronuclear injection of a construct composed of the ubiquitously-expressed *pCAG* promoter upstream of a *LoxP-GFP-STOP-LoxP* cassette and a *Neurog3* cDNA-IRES*-β-galactosidase* cassette (**S2A Fig**). We obtained two founder lines, the resulting transgenic animals being healthy, fertile, and not exhibiting any premature death (data not shown). N3OE animals were subsequently mated with HNF1b-CreER animals to generate HNF1b-CreER::N3OE (HNFN3OE—**S2B Fig**) double transgenic mice. These were initially treated for a short duration (two weeks) with Tam by oral gavage before being sacrificed. By means of X-Gal staining performed on pancreatic sections, we detected β-galactosidase activity exclusively within the ductal epithelium and solely upon tamoxifen treatment (**Fig 1A and 1B**). Quantitative RT-PCR analyses assaying the expression of *Neurog3* in the whole pancreas of these adult HNFN3OE mice confirmed the presence of *Neurog3* transcripts even after such a short induction time (**Fig 1C)**. Due to previous reports revealing important differences between *Neurog3* transcript and protein contents [16–18], Neurog3 protein expression had to be confirmed at adult ages in our inducible system. Thus, using immunohistochemical analyses targeting Neurog3 on pancreata of animals treated with Tam for only 2 weeks, we observed few ductal cells ectopically expressing *Neurog3* (**Fig 1E**). As expected, pancreata from untreated age-matched animals were found negative for Neurog3 (**Fig 1D**). Together, our results indicated that HNFN3OE double transgenic animals allowed the inducible misexpression of *Neurog3* specifically in ductal cells.

### *Neurog3* misexpression in ductal cells promotes the reactivation of the endocrine differentiation program

**Fig 2:**
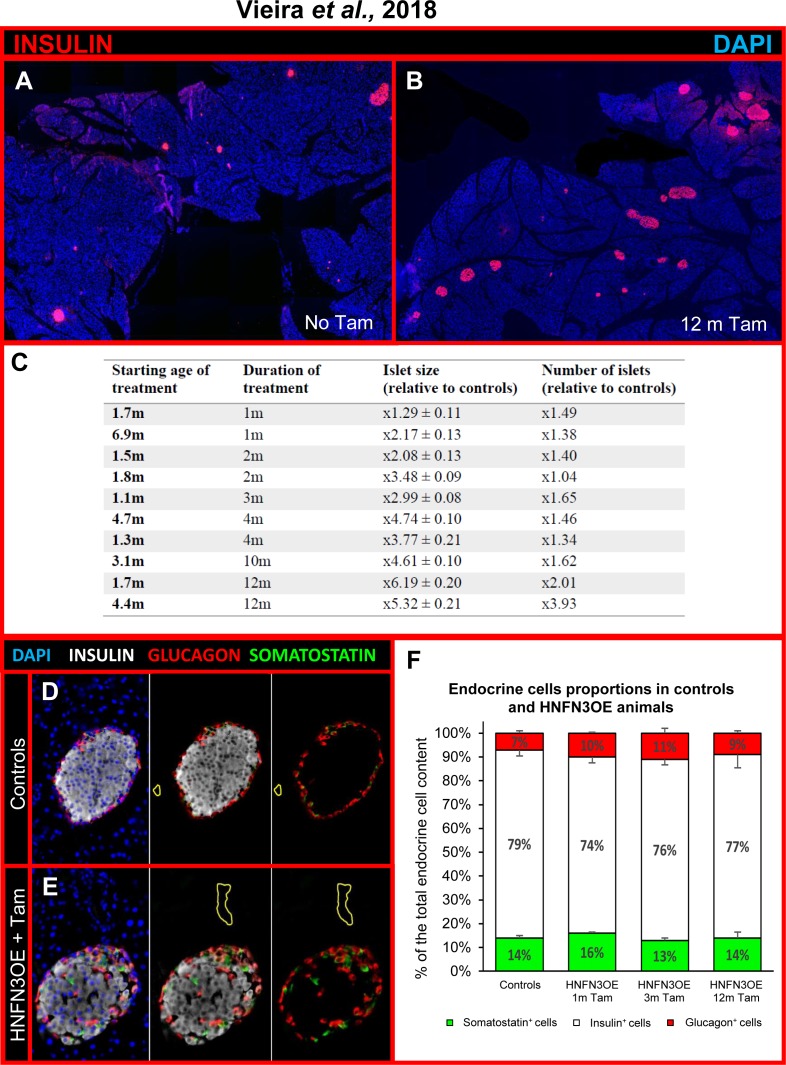
The ectopic expression of *Neurog3* triggered in adult pancreatic ductal cells results in hyperplastic islets with remodeled endocrine cell distribution but unchanged relative ratios. (**A-B**) Mosaic photographs of pancreatic sections of control (**A**) and HNFN3OE animal treated with Tam for 12 months (**B**) and assayed for insulin expression using immunohistochemical detection. Note the increase in islet number and size in Tam-treated HNFN3OE animal (**B**) compared to vehicle-administered control (**A**). Control in **A** is age-matched with **B**. (**C**) HNFOE littermates were treated with Tamoxifen for the indicated duration at the specified starting ages. The islets size and number were then analyzed for each group and compared to age-matched controls. Note the progressive islet hypertrophy and increase in islet count, these depending on the duration of Tam administration. The ratio displayed in the table indicate the relative increase in islet surface and count ± SEM, n = 3 per group. (**D-E**) Immunostaining of islets of Langerhans from vehicle- (**D**) and Tam- (**E**) treated pancreata displaying the distribution of insulin-, glucagon- and somatostatin-expressing cells. The typical islet organization observed in controls, with a core of β-cells surrounded by non β-cells (**D**), appears altered in Tam-treated animals (**E**), with a preferential localization of α- and δ-cells at one pole of the islets, in proximity of ducts (**E, S1 Movie**). For clarity, the ductal lumen is outlined with yellow lines. (**F**) Relative distribution of the insulin-, glucagon-, and somatostatin-producing cell populations in Tam-treated HNFN3OE pancreata compared to controls (Controls n = 6, 1m n = 4, 3m n = 3, 12m n = 3). Interestingly, the increases in insulin-, glucagon-, and somatostatin-producing cell counts are found to be similar in Tam-treated HNFN3OE animals, their relative proportions remaining unchanged when compared to controls. Statistics were performed using the Mann-Whitney test or unpaired t-test with Welch’s correction.

Additional tests established that providing Tam in drinking water also resulted in successful induction thereby allowing longer periods of treatment with diminished stress for the animals compared to oral gavage. We therefore treated HNFN3OE animals *per os* with Tam dissolved in drinking water for periods ranging from 1 up to 12 months. Importantly, a significant islet hypertrophy was observed in all treated animals (**Fig 2B and 2C**) compared to age-matched untreated or vehicle-administered counterparts (**Fig 2A**). Furthermore, a strong increase in the number of islets was detected in Tam-treated animals, suggestive of islet neogenesis (**Fig 2A–2C**). Quantitative immunohistochemical analyses confirmed these observations, the islet hypertrophy and islet multiplication reaching 5.3-fold and 3.9-fold, respectively, in animal treated with Tam for 12 months as compared to controls. Of note, these increases appeared to be dependent on the duration of Tam treatment, rather than on the age of Tam induction (**Fig 2C, S1 Movie**), suggesting that ageing is not limiting these neogenesis processes. Interestingly, closer examination of the hyperplastic islets not only revealed an augmented insulin^+^ cell count, but also a progressing increase in glucagon^+^ and somatostatin^+^ cell contents (**Fig 2D and 2E, S3A Fig, S1 Movie).** Interestingly, upon further analyses, the augmentations in number of insulin^+^, glucagon^+^, and somatostatin^+^ cells were found to be similar. Indeed, comparable and proportional increases for each endocrine cell subpopulation were noted, regardless of the duration of induction, resulting in islets displaying similar relative proportions of the different endocrine cell populations when compared to controls (**Fig 2F**).

**Fig 3:**
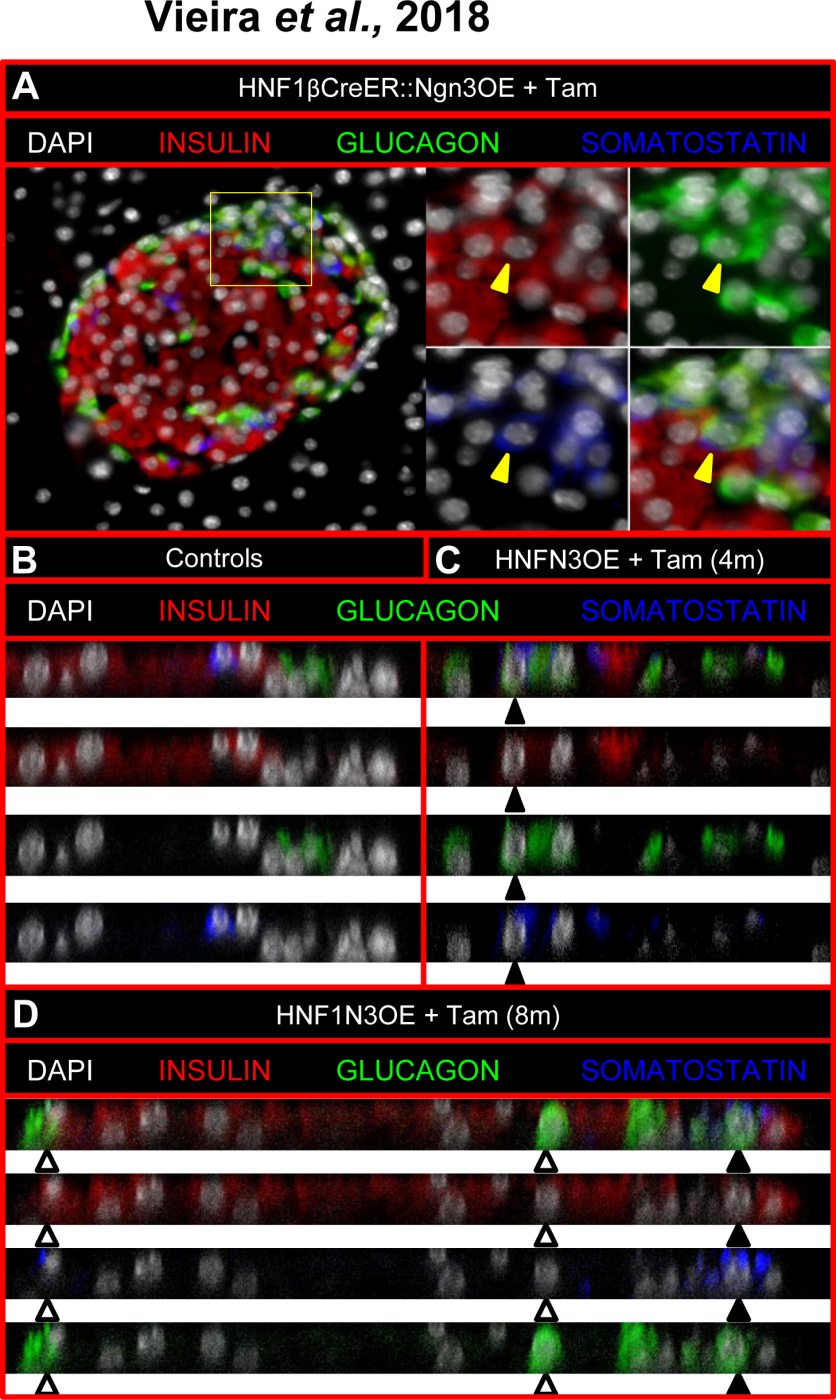
Observation of double and triple hormone-positive cells in adult Tam-treated HNFN3OE animals. (**A-D**) The examination of adult Tam-treated HNFN3OE pancreata using confocal analyses outlines an abnormal localization of non β-cells, with a clustering of such cells at a pole of the islets close to ducts (**A**, **Fig 2D and 2E**, **S1 Movie**). The analysis of such cell clusters assessed using orthogonal projection of a Z-stack shows cells co-expressing two (white arrowheads) or even three (black arrowheads) endocrine hormones (**C, D**), as seen during pancreas morphogenesis. As expected, such multi-hormone-expressing cells were not detected in untreated controls (**B**).

These concomitant augmentations in the numbers of the major endocrine cell subtypes were consistent with a re-enactment of the pancreatic developmental program controlled by *Neurog3*. Accordingly, as seen during pancreas morphogenesis, we observed a significant increase in the expression of *IA1*, one of the main target genes of Neurog3, the expression of other targets, such as *NeuroD1*, *Rfx6* and *Myt1*, appearing unchanged (**S3B Fig—**[19–22]). Along the same line, a few endocrine cells expressing multiple pancreatic hormones were detected (**Fig 3A–3D**), some of which expressing insulin and glucagon or insulin, glucagon, and somatostatin. Albeit rare, such cells were only present in our Tam-induced transgenic animals and, as expected, never detected in control adult animals (**Fig 3B**). It is worth reminding that multi-hormonal cells are normally solely observed during pancreas morphogenesis from embryonic (E) day E8.25 concomitantly with the first wave of endocrine development [16], such cells not contributing to the final endocrine cell population [13,23]. Should ductal cells represent the source of the neo-generated endocrine cells, one would expect either a progressive depletion of the ductal cell pool or a replacement of putatively converted cells. Interestingly, the total surface of the ductal epithelium was found unchanged comparing vehicle- (**S4A Fig**) and Tam- (**S4B Fig**) treated animals (8,26±1,2x10^-3^ mm^2^ of DBA^+^ cells per mm^2^ of pancreas in control animals vs 5,3±2,5x10^-3^ mm^2^ of DBA^+^ cells per mm^2^ of pancreas in HNFOE animals treated for 3 months with Tam—**S4E Fig**). Similarly, using long-term (10 days) 5-bromo-2'-deoxyuridine (BrdU) labeling to detect cells undergoing or having undergone replication, no difference was observed assessing vehicle- (**S4C Fig**) and Tam- (**S4D Fig**) treated pancreata (2,62±0,42 of BrdU^+^ cells per mm^2^ of pancreas in controls vs 2,96±0,24 of BrdU^+^ cells per mm^2^ of pancreas in HNFOE animals treated for 3 months with Tam—**S4E Fig**). Thus, should ductal cell be converted into endocrine cells upon *Neurog3* misexpression, our results would indicate that the overall ductal cell numbers remain unchanged, supporting the notion of slow compensatory mechanisms that cannot be captured even using relatively long durations to label proliferating cells. Altogether, our findings suggest that the misexpression of *Neurog3* in ductal cells induces the reactivation of the endocrine developmental program, resulting in the emergence of immature cells and the subsequent neogenesis of mature endocrine cells, the latter being allocated to the different endocrine cell lineages following the ratios normally underlying endocrine development.

### Ductal cells are continuously converted into endocrine cells upon *Neurog3* misexpression

**Fig 4:**
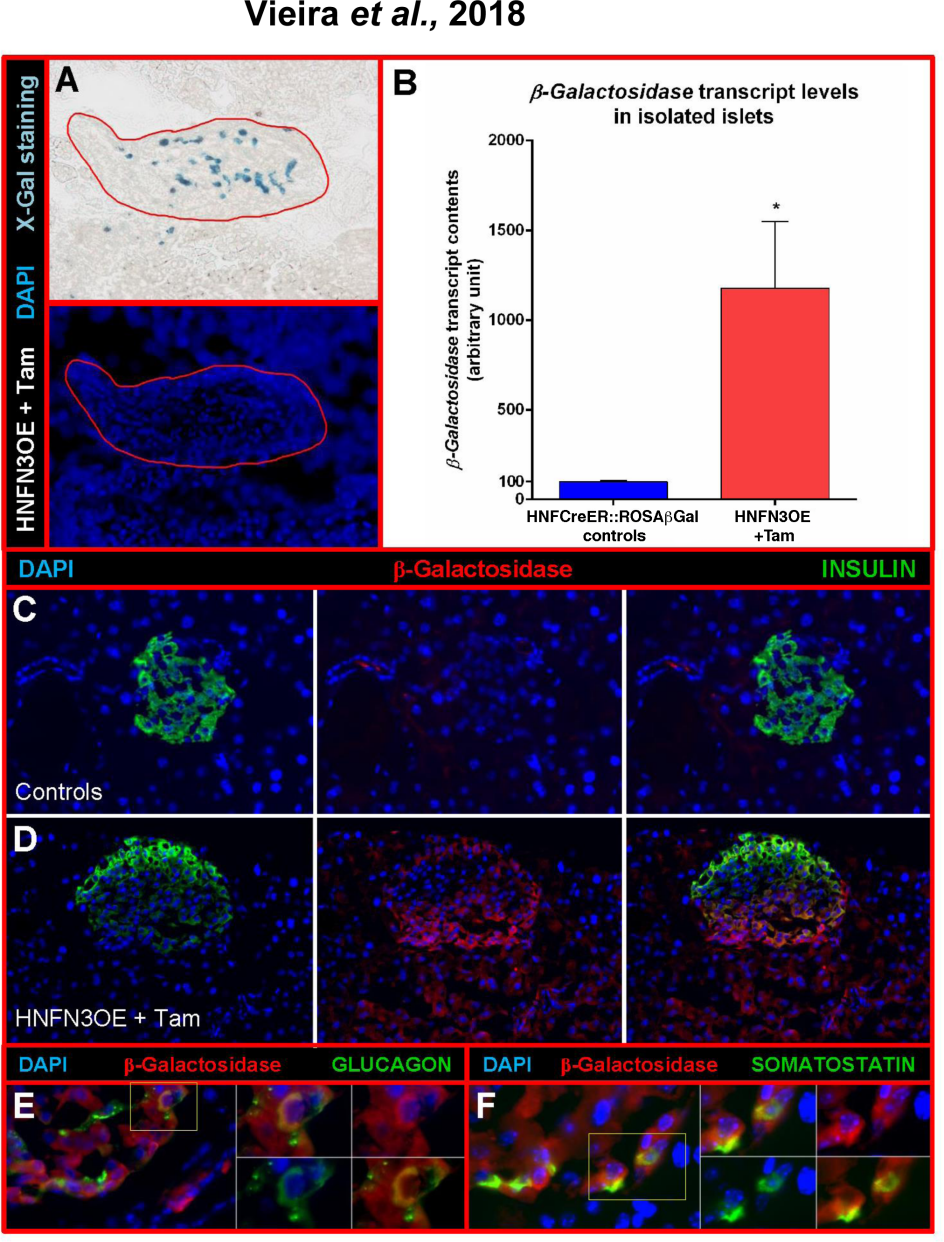
Lineage tracing experiments unravel the conversion of ductal cells into endocrine cells upon the sole *Neurog3* misexpression. (**A**) Taking advantage of the β-galactosidase tracer, we monitored the fate of the ductal cells ectopically expressing *Neurog3*. X-Gal staining reveals β-galactosidase-positive cells (previously ductal cells) within the islet of Langerhans (outlined with red lines) of Tam-treated HNFN3OE mice. (**B**) Quantitative RT-PCR analyses confirm the presence of *β-galactosidase* mRNA in the transcriptome of islets isolated from Tam-treated animals (n = 6 animals for each condition). Statistics were performed using the Mann-Whitney test (**C-D**) Immunohistochemical analyses combining β-galactosidase and insulin detection. While control pancreata are negative for β-galactosidase (**C**), their Tam-treated counterparts display cells positive for both insulin and β-galactosidase (**D**), indicating duct-to-endocrine cell conversion. Note that the apparent staining in the exocrine tissue is artefactual and caused by the antibody used. (**E-F**) Accordingly, several glucagon^+^ (**E**) or somatostatin^+^ (**F**) cells are also found labeled with the ductal cell tracer, β-galactosidase.

**Fig 5:**
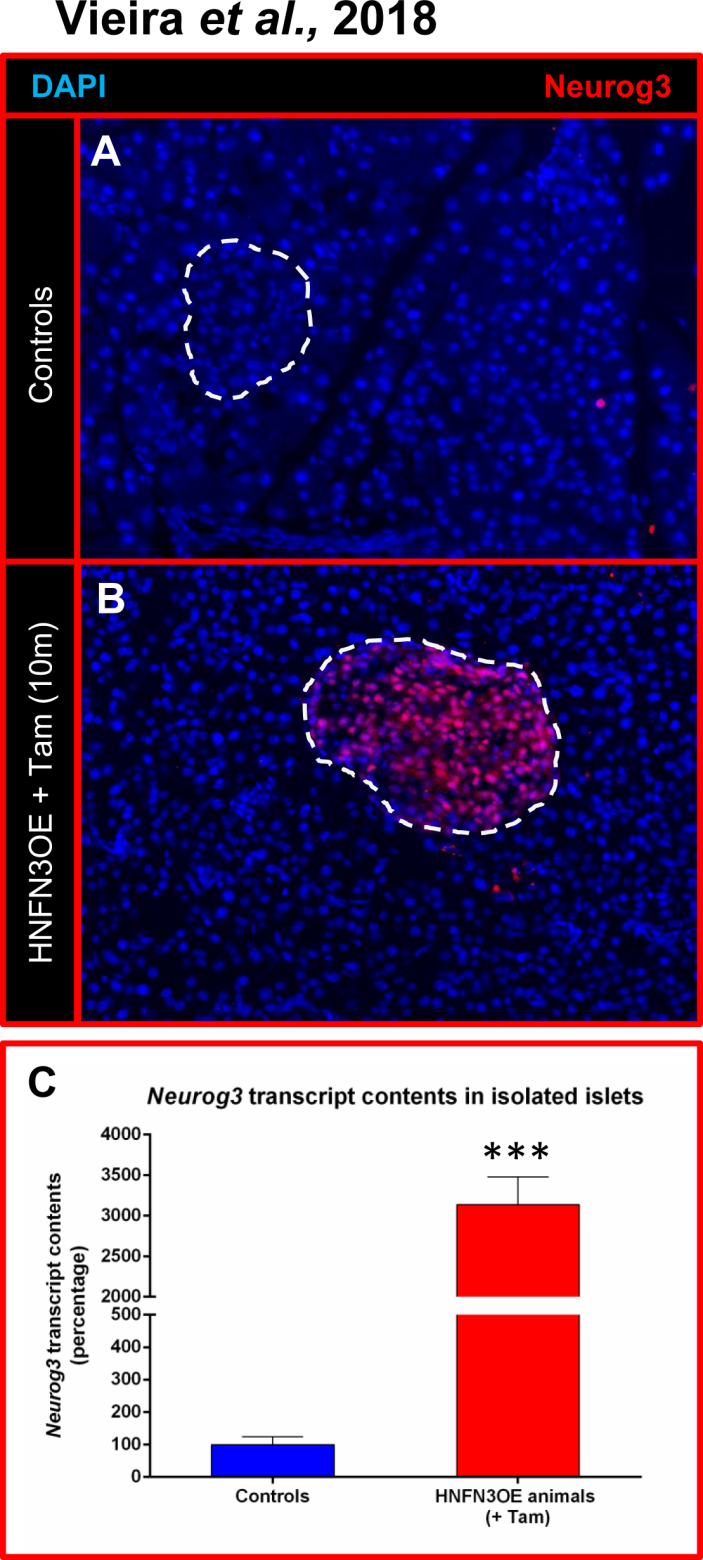
*Neurog3* misexpression is maintained in newly-formed endocrine cells in adult Tam-treated HNFN3OE pancreata. The observation of islets in HNFN3OE animals treated with Tam for 10 months demonstrates that a clear majority of endocrine cells misexpress Neurog3 (**A-B**), this result being confirmed by qPCR analyses (**C**, n = 6 for each condition) Statistics were performed using the Mann-Whitney test.

To characterize the islet composition of HNFN3OE pancreata, we further analyzed their endocrine cell populations compared to controls. In the islets of Langerhans of control animals, endocrine non-β-cells are classically distributed within the islet mantle area without any specific pattern (**Fig 2D**). Interestingly, in our transgenic animals, we observed an accumulation of α- and δ-cells in areas of the islet situated close to ducts (**Fig 2E, S1 Movie**), suggestive of a putative ductal origin. To assess the origin of the newly formed endocrine cells and thereby determine whether these truly exhibited a ductal ontogeny, we took advantage of the lineage tracer present in our model, *β-galactosidase* (**S2B Fig**). We first performed X-Gal staining to assess β-galactosidase activity. Interestingly, unlike in control islets (**Fig 1A**), we observed β-galactosidase^+^ cells (of ductal origin) within Tam-treated HNFN3OE islets (**Fig 4A**). Accordingly, in isolated islets of Langerhans from HNFN3OE animals (following 6 months of Tam treatment), we noted a 11.78±3.72-fold increase in *β-galactosidase* transcript contents (**Fig 4B**). Furthermore, by means of immunohistochemical analyses, we demonstrated that a high number of insulin-producing cells of induced HNFN3OE animals were positive for the β-galactosidase ductal cell tracer (**Fig 4D**), while control sections displayed no β-galactosidase expression (**Fig 4C**). Similarly, glucagon^+^ or somatostatin^+^ cells were found to be positive for β-galactosidase (**Fig 4E and 4F**). Lastly, the assessment of Neurog3-labelled endocrine cells confirmed that a vast majority of islet cells expressed Neurog3 following 10 months of Tam administration (**Fig 5A and 5B**). This was further confirmed by quantitative RT-PCR analysis demonstrating a cumulative 31.4±3.4-fold increase in *Neurog3* transcript levels in islets from HNFN3OE animals compared to their control counterparts (**Fig 5C**). Together, these findings indicate that the misexpression of *Neurog3* in ductal cells results in their eventual conversion into endocrine cells, supporting the notion of a recapitulation of the developmental endocrine differentiation program at an adult age.

### The maintained expression of *Neurog3* in newly-generated endocrine cells does not alter their function

**Fig 6:**
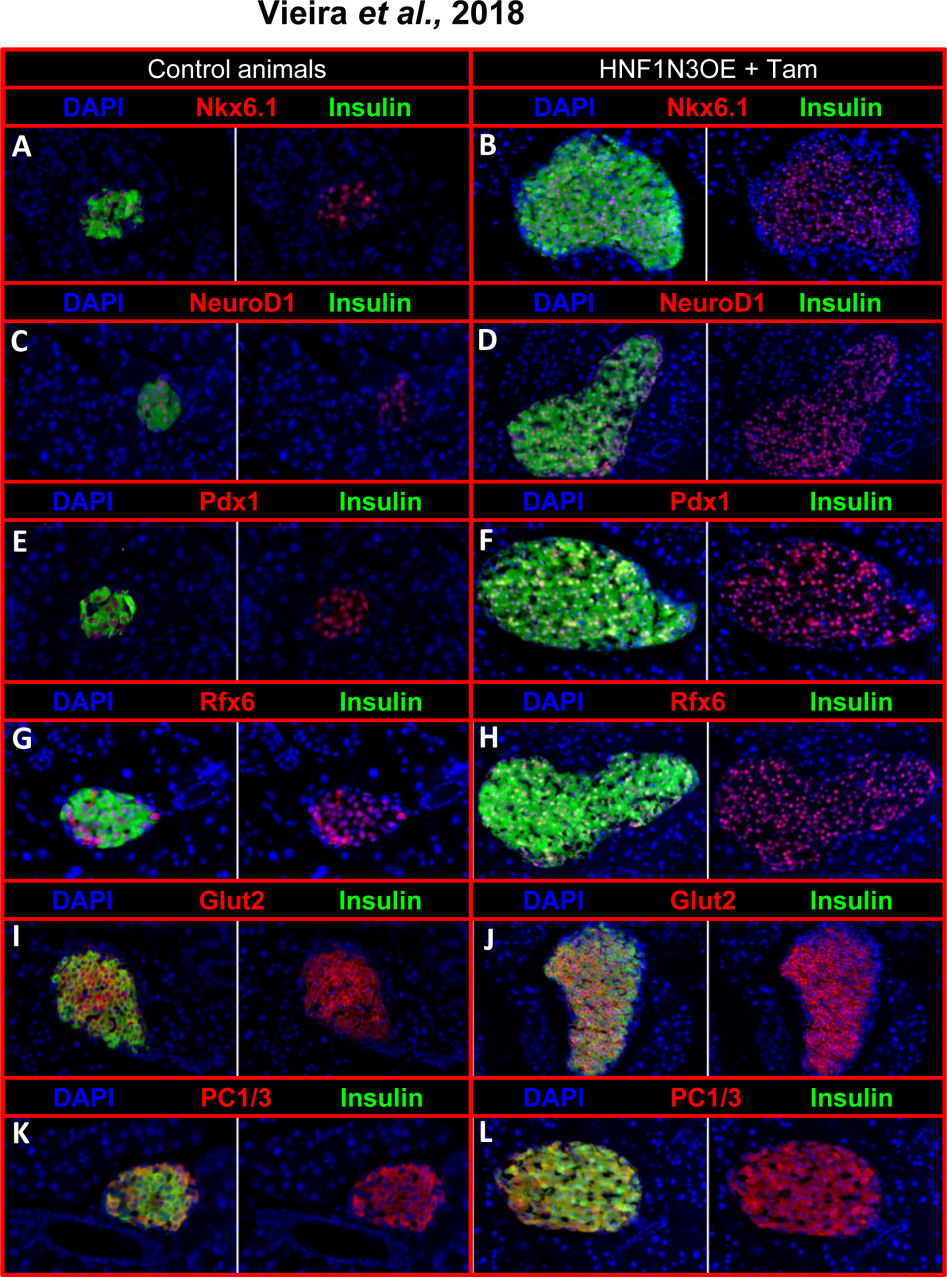
Phenotypical analyses of islet cells from Tam-treated HNFN3OE pancreata. Representative pictures of immunohistochemical analyses performed on pancreas sections from untreated (**A, C, E, G, I, K**) and age-matched Tam-treated HNFN3OE mice (**B, D, F, H, J, L**) using the indicated antibody combinations. All insulin^+^ cells (preexisting and neogenerated) express the *bona fide* β-cell markers Nkx6.1 (**A-B**), NeuroD1 (**C-D**), Pdx1 (**E-F**), Rfx6 (**G-H**), Glut2 (**I-J**), and PC1/3 (**K-L**).

**Fig 7:**
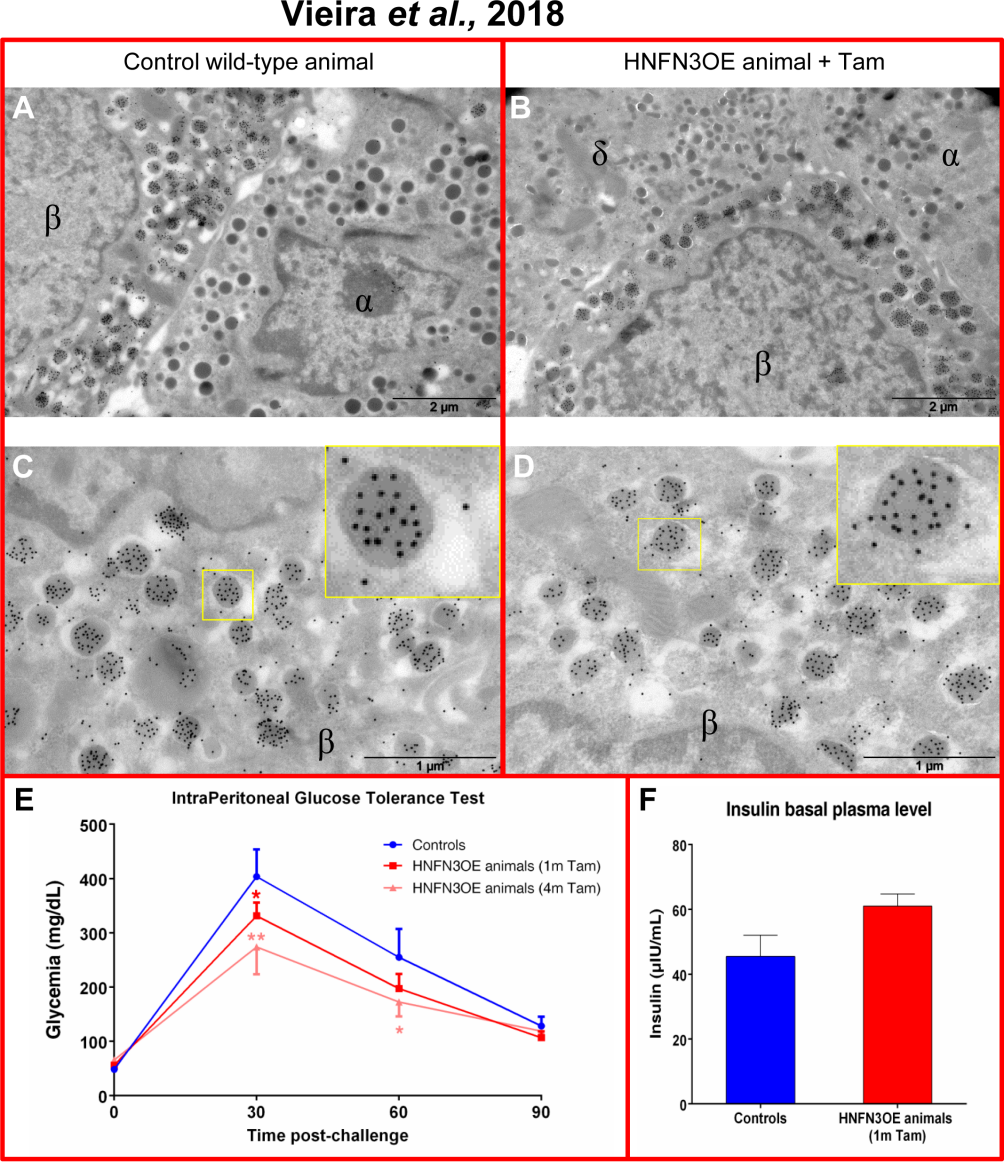
HNFN3OE animals display a normal endocrine cell ultrastructure and exhibit an improved response upon glucose stimulation *in vivo*. (**A-D**) Electron microscopy examinations (n = 3, 200 photographs analyzed per sample) reveal no difference in the ultrastructure of endocrine cells comparing control (**A**) and transgenic animals (**B**). Focusing on insulin-producing cells combining electron microscopy and immuno-gold labelling of insulin, all cells presenting a β-cell ultrastructure are found positive for insulin. Similarly, all cells labeled with insulin exhibit a β-cell ultrastructure (**C-D** and insets). (**E**) Upon being subjected to intraperitoneal glucose tolerance tests, HNFN3OE animals treated with Tam for one month display a lower peak in glycemia and a faster return to normoglycemia compared to their control counterparts. This improved tolerance is further enhanced following 4 months of Tam administration (Controls n = 5, 1m n = 5, 4m n = 4). (**F**) Assessment of basal insulin serum levels in HNFN3OE animals treated with Tam or not, indicating a normal basal insulin secretion despite an increased β-like cell mass (n = 3 for each condition). Statistics were performed using the Mann-Whitney test.

*Neurog3* expression is normally solely detected during embryogenesis, its expression being strongly down-regulated once endocrine cells have been generated [24]. In addition, Neurog3 was previously demonstrated to have a very short half-life [25]. One may therefore wonder whether the maintained expression of *Neurog3* in neo-generated endocrine cells may alter their function (**Fig 5A and 5B**). To address this question, we first performed thorough immunohistochemical analyses of endocrine marker genes on pancreata from HNFN3OE animals treated for 10 months with Tam. Importantly, all insulin-producing cells (that is, pre-existing and supplementary/neo-generated ones) were found positive for the *bone fide* β-cell markers, including Nkx6.1 (**Fig 6A and 6B**), NeuroD1 (**Fig 6C and 6D)**, Pdx1 (**Fig 6E and 6F)**, Rfx6 (**Fig 6G and 6H)**, Glut-2 (**Fig 6I and 6J)**, and PC1/3 (**Fig 6K and 6L**), indicating that the Neurog3^+^ β-like cells expressed the same markers as their endogenous counterparts. Accordingly, in a continued effort to ascertain that all insulin-producing cells displayed a β-cell phenotype, we used electron microscopy combined with insulin detection by means of immuno-gold labeling. These thorough analyses (n = 3, 200 photographs examined per condition) allowed us to demonstrate that almost all cells displaying a β-cell ultrastructure secreted insulin and that almost all cells producing insulin exhibited a β-cell morphology (**Fig 7A–7D**). Aiming to determine whether neo-generated insulin^+^ cells were physiologically functional, we subjected HNFN3OE animals to intraperitoneal glucose tolerance tests (IPGTTs). Importantly, HNFN3OE mice treated for only 1 month with Tam displayed an improved response to the glucose bolus with a lower peak in glycemia and a faster return to euglycemia (**Fig 7E**), this without alteration of basal fasted insulin secretion (**Fig 7F**). These results support the notion of an increased β-like cell mass in animals misexpressing *Neurog3* in ductal cells. As interesting were the results obtained for animals that had been exposed for 4 months to Tam with an even more remarkable and improved response to IPGTT (**Fig 7E**), consistent with the further increased number of functional insulin-positive cells observed in the pancreata of these animals compared to controls (**Fig 2C**).

**Fig 8:**
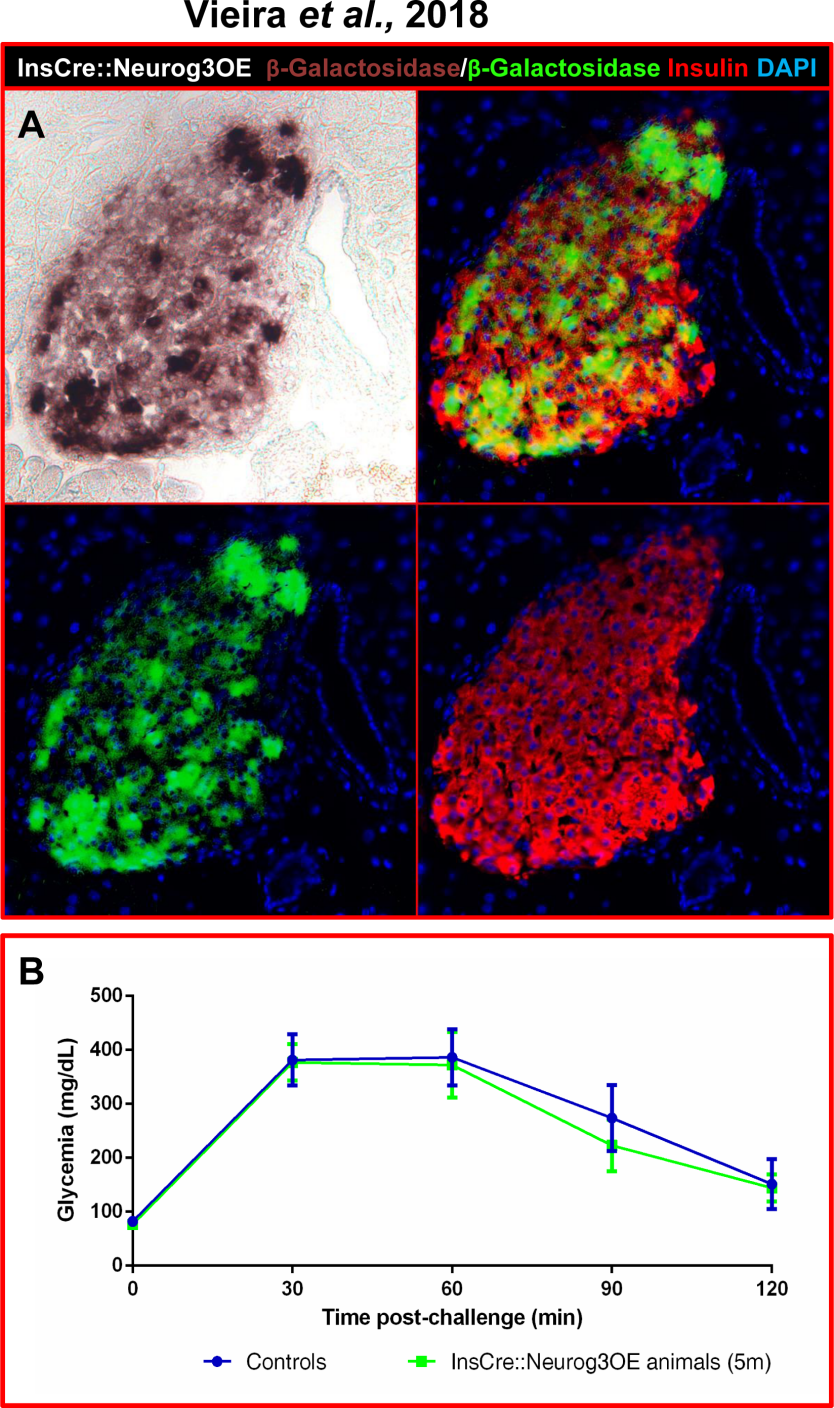
*Neurog3* misexpression in insulin^+^ cells does not alter β-cell function. (**A**) A combination of immunohistochemical detection and Salmon-Gal staining was used to assess β-galactosidase activity in InsCre::Neurog3OE pancreata. 80% of insulin-producing cells appeared positive for β-galactosidase. For clarity, Salmon-Gal staining was converted to a green labeling in the photographs. (**B**) Intraperitoneal glucose tolerance tests of adult InsCre::Neurog3OE animals (5-month old, n = 5) do not show any difference in glucose response comparing transgenic animals and their age-matched transgene-negative littermates (n = 4), indicating that maintained *Neurog3* misexpression in β-cells does not impair their ability to secrete insulin in response to glucose stimulation. Statistics were performed using the Mann-Whitney test.

In order to definitely exclude any deleterious effect of sustained *Neurog3* expression in adult β-cells, we generated and analyzed InsCre::Neurog3OE mice by crossing Insulin-Cre animals (harboring a transgene in which the *insulin* promoter drives the expression of the *Cre recombinase—*[13] with Neurog3OE mice (**S2C Fig**). Interestingly, despite a previous report indicating that mice expressing *Neurog3* under the control of the *insulin* promoter would die shortly after birth due to hyperglycemia [26], InsCre::Neurog3OE double transgenics were viable, fertile, healthy, and displayed normal basal glycemia (data not shown). We subsequently evaluated the efficiency of *Neurog3* misexpression in β-cells using Salmon-Gal staining. Our results revealed β-galactosidase activity within the core of the islets in approximately 80% of insulin-positive cells (**Fig 8A**), thereby demonstrating the efficiency of this double transgenic line and thus, the misexpression of *Neurog3* in insulin-expressing cells. Importantly, upon IPGTT in 5-month old InsCre::Neurog3OE animals, we observed a similar response to that of the transgene-negative littermates (**Fig 8B**), indicating that InsCre::Neurog3OE animals retained the ability to restore euglycemia upon glucose stimulation despite continuous *Neurog3* expression in mature and functioning β-cells, thus further validating the observations made in HNFN3OE animals. Together, our results indicate that the maintained expression of *Neurog3* in neogenerated β-like cells does not alter their function, such cells secreting insulin and being able to counter the consequences of glucose-induced hyperglycemia.

## Discussion

The question of the ability of ductal cells to adopt an alternative pancreatic phenotype *in vivo* has been discussed in recent years, some reports presenting evidence of a ductal contribution during endocrine cell regeneration [6,8,10–12,14,27–31], while others excluded it altogether [7,9,32–35]. However, the *in vivo* approaches previously used mostly relied on diverse models of pancreas injury, resulting in different degrees of stress and, most likely, in proportional/adapted responses. Here, an alternative approach was adopted with the misexpression of the *Neurog3* gene in adult ductal cells to determine whether the adult pancreatic ductal population retained the inherent developmental capability of generating endocrine cells, outside of any pancreatic injury. Importantly, our study demonstrates that the sole ectopic expression of *Neurog3* in HNF1b^+^ ductal cells results in islet neogenesis and islet hypertrophy. As interesting was the finding that the islet hypertrophy was the consequence of proportional increases in insulin-, glucagon-, and somatostatin-producing cell counts, resulting in normal relative ratio when compared to controls. Lineage tracing experiments confirmed a ductal ontogeny for these supplementary endocrine cells. It is worth noting that the supplementary insulin^+^ cells presented similar phenotypical and ultra-structural characteristics to those of endogenous β-cells. These were functional with an improved response upon glucose stimulation. Further analyses demonstrated that the maintained misexpression of *Neurog3* in adult β-cells does not alter their function.

### Duct-to-endocrine cell conversion upon *Neurog3* misexpression

The generation of HNFN3OE double transgenic animals allowed the spatiotemporal control of the misexpression of *Neurog3* in ductal cells. The HNF1b-CreER mouse line, despite its relatively low efficiency, permitted us to achieve *Neurog3* misexpression specifically in the ductal epithelium. The most striking consequence observed following the long-term misexpression of *Neurog3* in adult ductal cells was the resulting significant and progressive increase in the number of all islet cells. In addition, an increase, even though limited, in the islet count was also noted, suggestive of islet neogenesis processes. Importantly, lineage tracing experiments revealed that the supernumerary endocrine cells originated from the ductal cells that re-expressed *Neurog3*. Using quantitative analyses, we observed that, although increased in numbers, the relative proportions of glucagon^+^, insulin^+^ and somatostatin^+^ cells observed in Tam-treated HNFN3OE pancreata were similar to that of controls, these being maintained independently of the duration of Tam induction. These results indicate that the processes leading to the generation of new endocrine cells in this adult context faithfully recapitulate those underlying the embryonic development of endocrine cells. Indeed, during development, Neurog3^+^ precursor cells eventually give rise to approximately 80±5% of β-cells, 14±2% of α-cells, and 6±5% of δ-cells. Accordingly, our findings indicate that the misexpression of *Neurog3* in ductal cells results in endocrine cell neogenesis respecting these ratios. The processes implicated in this adult context appear to re-enact those underlying islet cell genesis during embryogenesis with the mobilization of the Neurog3 targets, such as IA1, Rfx6, Myt1 [19,21,22]. However, our results appear to be in contradiction with a recent publication suggesting that *Neurog3* activation is not sufficient to direct duct-to-β-cell transdifferentiation in the adult pancreas [36]. In this study, the fate of Neurog3^+^ cells arising in the ducts of animals following alloxan injection or PDL was investigated. Traced *Neurog3*-re-expressing cells did not express pancreatic hormones, which led the authors to conclude that the activation of this gene alone was not sufficient to trigger a full duct-to-endocrine cell conversion. However, as mentioned above, pancreatic regeneration analyses hitherto provided contradictory results, the activated regeneration mechanisms seemingly being dependent on the injury model. For instance, additional recent experiments outlined a small increase in the number of ductal cells ectopically expressing *insulin* after the deletion of the *Fbw7* gene which is involved in the ubiquitination of Neurog3 [29], thus suggesting that the stabilization of Neurog3 could initiate duct-to-endocrine cell conversion. In the current approach, we conducted our analyses over several months (and up to a year), thereby allowing us to induce processes that could require time. Thus, by excluding the bias introduced by pancreatic injury, our long-term approach allowed us to unravel the intrinsic plasticity of duct cells allowing them to be reprogrammed into endocrine cells upon *Neurog3* misexpression.

Our results clearly demonstrate that pancreatic duct cells convert into mature endocrine cells upon *Neurog3* misexpression. However, one could wonder whether all HNF1b^+^ ductal cells have the potential to become endocrine cells or whether this plasticity only concerns a ductal subpopulation. While addressing these questions would require further work, one could envision an interesting compromise in which HNF1b^+^ cells could, under specific conditions, re-express *Neurog3* (as previously reported) and reactivate endocrine developmental processes. Ductal cells could therefore de-differentiate into *Neurog3*^*+*^ precursor cells that would then re-differentiate into endocrine cells.

As interesting is the question of the processes involved in the neo-generation of endocrine cells. Indeed, one could, for instance, wonder whether some of the newly-formed β-cells passed through a phase of glucagon or somatostatin expression prior to acquire a β-cell identity. The same question could in fact apply to all new endocrine cells as multi-hormone expressing cells were clearly detected in Tam-treated HNFN3OE pancreata. Obviously, addressing these interrogations would require much work with the establishment of transgenic animals both allowing the misexpression of *Neurog3* in ductal cells and the inducible lineage tracing of insulin-, glucagon-, or somatostatin-expressing cells.

Another interesting feature of our analyses was the apparent absence of modification in ductal cell counts. This finding was surprising as one would expect a depletion of the ductal cell pool concomitantly with the conversion of *Neurog3*-misexpressing ductal cells into endocrine cells. Since we did not observe such an alteration in ductal structure in transgenic animals, we presume that a replenishment of the ductal epithelium occurred. However, no significant difference was observed in the number of mitotic cells in the ductal epithelium of our transgenic animals compared to their WT counterparts. These findings do not exclude proliferation as a mechanism of restoration of the ductal epithelium, however our data point towards a relatively slow proliferation rate mirroring the slow increase in endocrine cell count.

### Sustained *Neurog3* expression in newly generated endocrine cells does not impair their functionality

*Neurog3* expression is normally shut down during pancreatic organogenesis as soon as endocrine cells initiate hormone expression, although a report suggested that the expression of *Neurog3* is not extinguished but extremely reduced [37]. Per this report, all mature endocrine cells express very low amounts of *Neurog3* mRNA in adults and appear to require this gene for proper insulin secretion. However surprising, these results are consistent with our findings and are in line with the observed maintenance of a functional and mature phenotype of newly generated endocrine cells despite the continued expression of *Neurog3*. Indeed, one would assume that the sustained expression of *Neurog3* observed in the islets of our transgenic animals could prevent the full differentiation of neo-generated endocrine cells, maintaining them in an immature state. To further evaluate the influence of continuous expression of *Neurog3* on insulin secretion, we therefore generated the InsCre::Neurog3OE double transgenic mouse line and confirmed, as seen in HNFN3OE animals, that insulin^+^ cells ectopically expressing *Neurog3* were phenotypically indistinguishable from their endogenous counterparts. Accordingly, HNFN3OE animals displayed a normal basal insulin serum concentration and demonstrated an appropriate, even quite improved, response to glucose stimulation. Similarly, while immature β-cells usually present lighter vesicles when assayed using electron microscopy, indicating a defect in insulin processing [38], our electron microscopy analyses demonstrated all the hallmarks of mature insulin secretory vesicles. Thus, although it has been reported that low levels of *Neurog3* transcripts in β-cells are necessary for proper insulin secretion [37], it appears that even higher expression levels of Neurog3 in adult endocrine cells do not influence their maturation or the secretory pathways of insulin-producing cells.

Taken together, our analyses demonstrate that (1) adult ductal cells retain the developmental capability to be converted into endocrine cells upon *Neurog3* misexpression following processes similar to those underlying embryogenesis; (2) Neurog3-induced endocrine cell neogenesis can be continued for several months; (3) the maintained ectopic expression of *Neurog3* in endocrine/insulin^+^ cells does not alter their phenotype nor function. Based on these findings, we suggest that ductal cells could represent a renewable source of new β-like cells and that strategies aiming at controlling the expression of *Neurog3*, or of its molecular targets/co-factors, could be considered in the context of finding new ways to generate insulin-secreting cells.

## Supporting information

S1 Movie Islet hypertrophy and preferential localization of glucagon-producing cells adjacent to ducts in HNFN3OE pancreata. Pancreata from 9-month old Tam-treated (for 8 months) HNFN3OE animals (Bottom) and from age-matched untreated controls (Top) were sectioned (8μm thickness) and every section was stained with anti-insulin and anti-glucagon antibodies and photographed. Using the Amira software (Mercury), the pictures were aligned to form a z-stack and movies of the resulting isosurface representations were generated (the ductal lumen being manually colored in blue). Note the preferential localization of glucagon-producing cells at a pole of the islet adjacent to ducts in Tam-treated HNFN3OE pancreata.
(MOV)

S1 Fig Analyses of the efficiency of the HNF1b-CreER mouse line. (**A**) Schematics depicting the generation of double transgenic animals expressing β-galactosidase specifically in HNF1b-positive pancreatic ductal cells. HNF1b-CreER mice (generated using a transgene encompassing the Tam-inducible Cre recombinase inserted within the first exon of the *HNF1b* gene) were mated with ROSA-β-gal animals, a well-established reporter line allowing β-galactosidase expression solely in Cre-producing cells. (**B-E**) The efficiency of the resulting HNF1b-CreER::ROSA-β-Gal line was assessed combining immunohistochemical detection (**B**) and X-Gal staining (**C-D**). Note the detection of numerous β-galactosidase^+^ cells in the DBA^+^ ductal cells of Tam-treated HNF1b-CreER::ROSA-β-Gal animals (**B**), such detection being confirmed by X-Gal staining (**C-D**). Quantitative immunohistochemical analyses support these results with a labeling of 47±18% of ductal cells with β-galactosidase (**E**) (n = 6 for controls and n = 12 for transgenic animals). Statistics were performed using one sample t-test.
(TIF)

S2 Fig Description of the transgenic lines used and of the expected genetic modifications (see the main text for details). (TIF)

S3 Fig Quantification of the different endocrine cell populations in Tam-treated HNFN3OE animals. (**A**) HNFN3OE mice were treated with Tam for the indicated increasing durations (same groups of mice as in **Fig 2C**). Quantitative immunohistochemical analyses were used to assay the different endocrine cell populations (focusing on insulin-, glucagon-, and somatostatin-expressing cells): a progressive increase for all islet cell subtypes is observed in Tam-administered transgenics as compared to age-matched untreated controls, such augmentations progressing with the duration of Tam exposure. Statistics were performed using the Mann-Whitney test or unpaired t-test with Welch’s correction (**B**) Quantitative RT-PCR analyses assessing the expression of known *Neurog3* target genes in adult Tam-treated HNFN3OE pancreata versus controls, demonstrating a significant increase in *IA1* transcript levels, while *NeuroD1*, *Rf6*, and *Myt1* expressions are non significantly increased (n = 3 for each condition). Statistics were performed using the Mann-Whitney test.
(TIF)

S4 Fig Maintenance of the ductal cell population in adult Tam-treated HNFN3OE pancreata. Using quantitative immunohistochemical analyses comparing ductal cells in HNFN3OE pancreata treated with vehicle (**A**) or Tam (**B**) for 12 months, no difference was detected in the number of ductal cells. Similarly, using long-term BrdU labelling (10 days prior to sacrifice), the numbers of proliferating ductal cells were found unchanged comparing vehicle- (**C**) and Tam-(**D**) treated animals (no significant difference was noted counting the numbers of BrdU^+^ or DBA^+^ ductal cells in both conditions). Ductal epithelium surface and proliferation were assessed comparing untreated animals and HNFOE Tam-treated for 3 months (**E**), with no significant difference observed. Statistics were performed using the Mann-Whitney test or unpaired t-test with Welch’s correction.
(TIF)
