# Supplementary figures and images for: Neurog3 misexpression unravels mouse pancreatic ductal cell plasticity
Source: PLoS One. 2018 Aug 9;13(8):e0201536. doi: 10.1371/journal.pone.0201536 (PMC6084906; doi:10.1371/journal.pone.0201536)

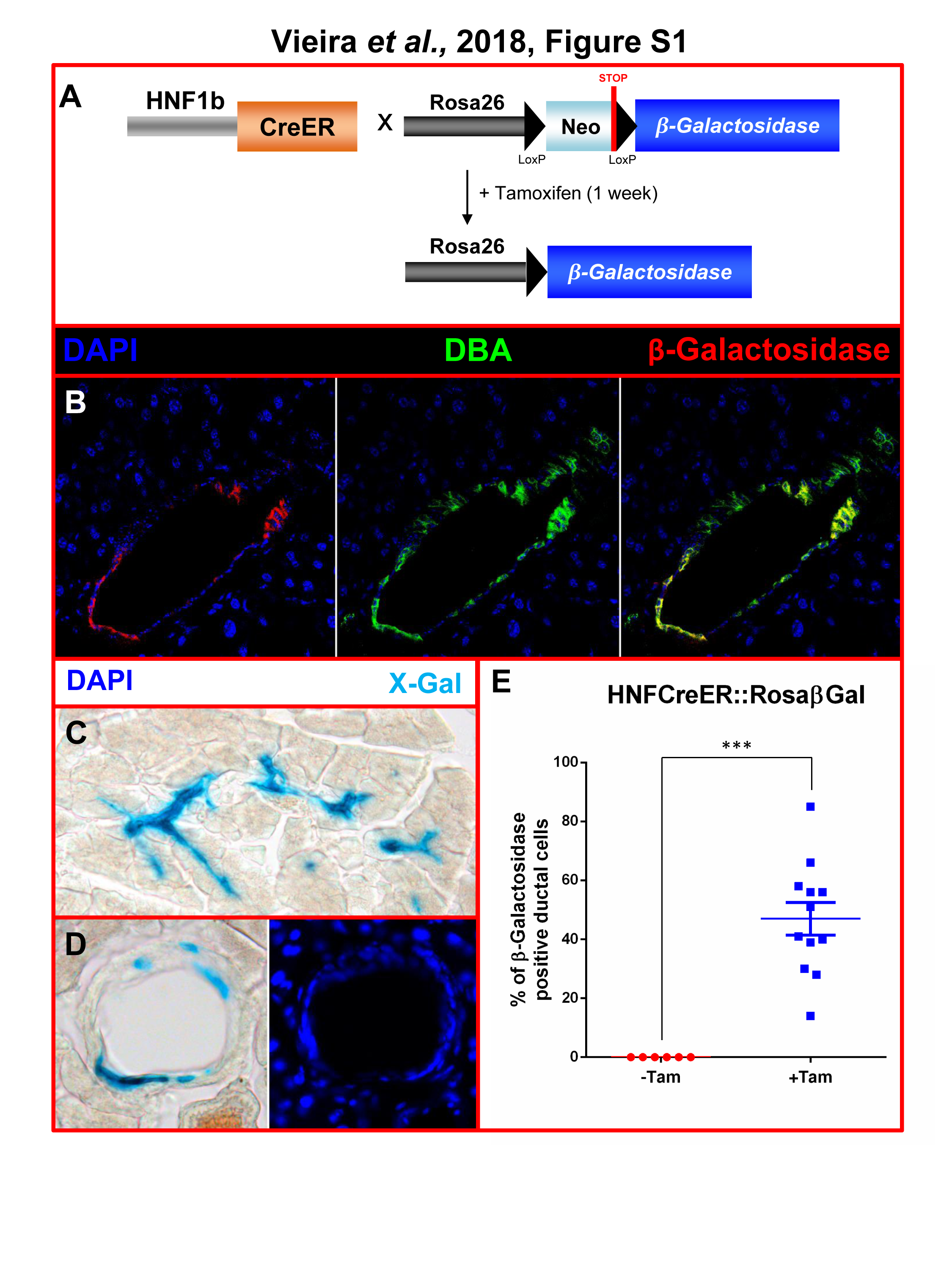

Supplement: S1 Fig — (A) Schematics depicting the generation of double transgenic animals expressing β-galactosidase specifically in HNF1b-positive pancreatic ductal cells. HNF1b-CreER mice (generated using a transgene encompassing the Tam-inducible Cre recombinase inserted within the first exon of the HNF1b gene) were mated with ROSA-β-gal animals, a well-established reporter line allowing β-galactosidase expression solely in Cre-producing cells. (B-E) The efficiency of the resulting HNF1b-CreER::ROSA-β-Gal line was assessed combining immunohistochemical detection (B) and X-Gal staining (C-D). Note the detection of numerous β-galactosidase+ cells in the DBA+ ductal cells of Tam-treated HNF1b-CreER::ROSA-β-Gal animals (B), such detection being confirmed by X-Gal staining (C-D). Quantitative immunohistochemical analyses support these results with a labeling of 47±18% of ductal cells with β-galactosidase (E) (n = 6 for controls and n = 12 for transgenic animals). Statistics were performed using one sample t-test. (TIF) [file pone.0201536.s002.tif]

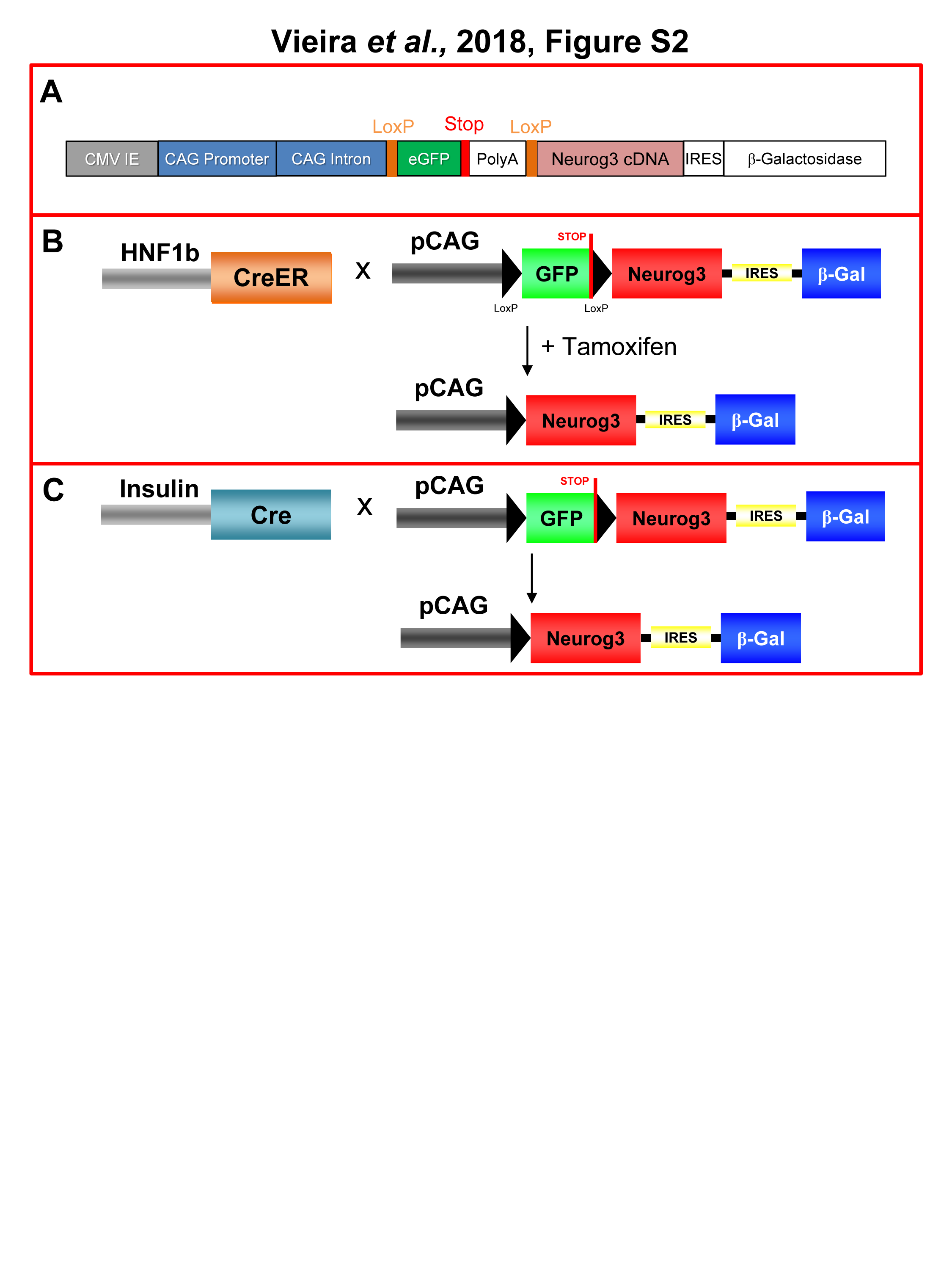

Supplement: S2 Fig — (TIF) [file pone.0201536.s003.tif]

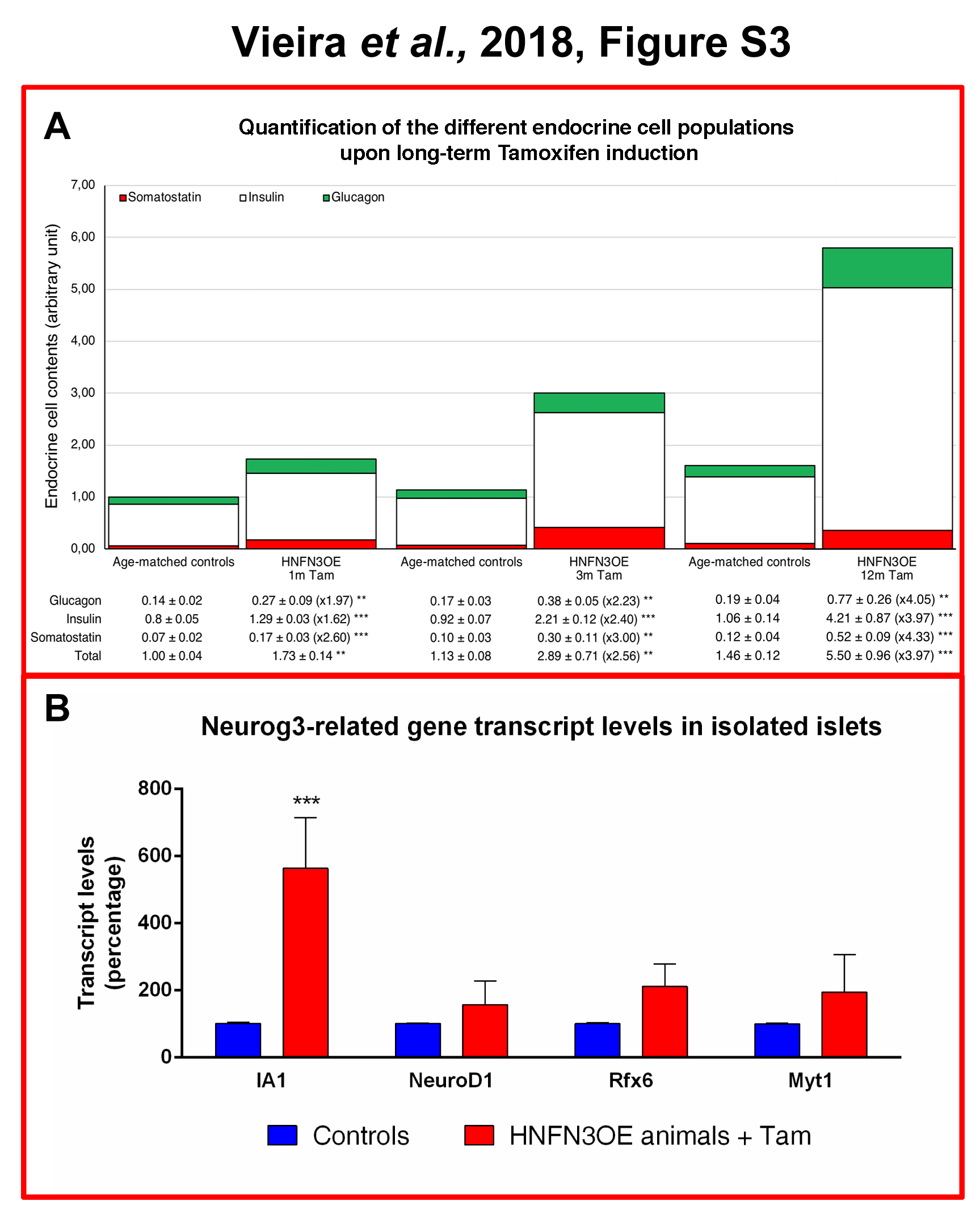

Supplement: S3 Fig — (A) HNFN3OE mice were treated with Tam for the indicated increasing durations (same groups of mice as in Fig 2C). Quantitative immunohistochemical analyses were used to assay the different endocrine cell populations (focusing on insulin-, glucagon-, and somatostatin-expressing cells): a progressive increase for all islet cell subtypes is observed in Tam-administered transgenics as compared to age-matched untreated controls, such augmentations progressing with the duration of Tam exposure. Statistics were performed using the Mann-Whitney test or unpaired t-test with Welch’s correction (B) Quantitative RT-PCR analyses assessing the expression of known Neurog3 target genes in adult Tam-treated HNFN3OE pancreata versus controls, demonstrating a significant increase in IA1 transcript levels, while NeuroD1, Rf6, and Myt1 expressions are non significantly increased (n = 3 for each condition). Statistics were performed using the Mann-Whitney test. (TIF) [file pone.0201536.s004.tif]

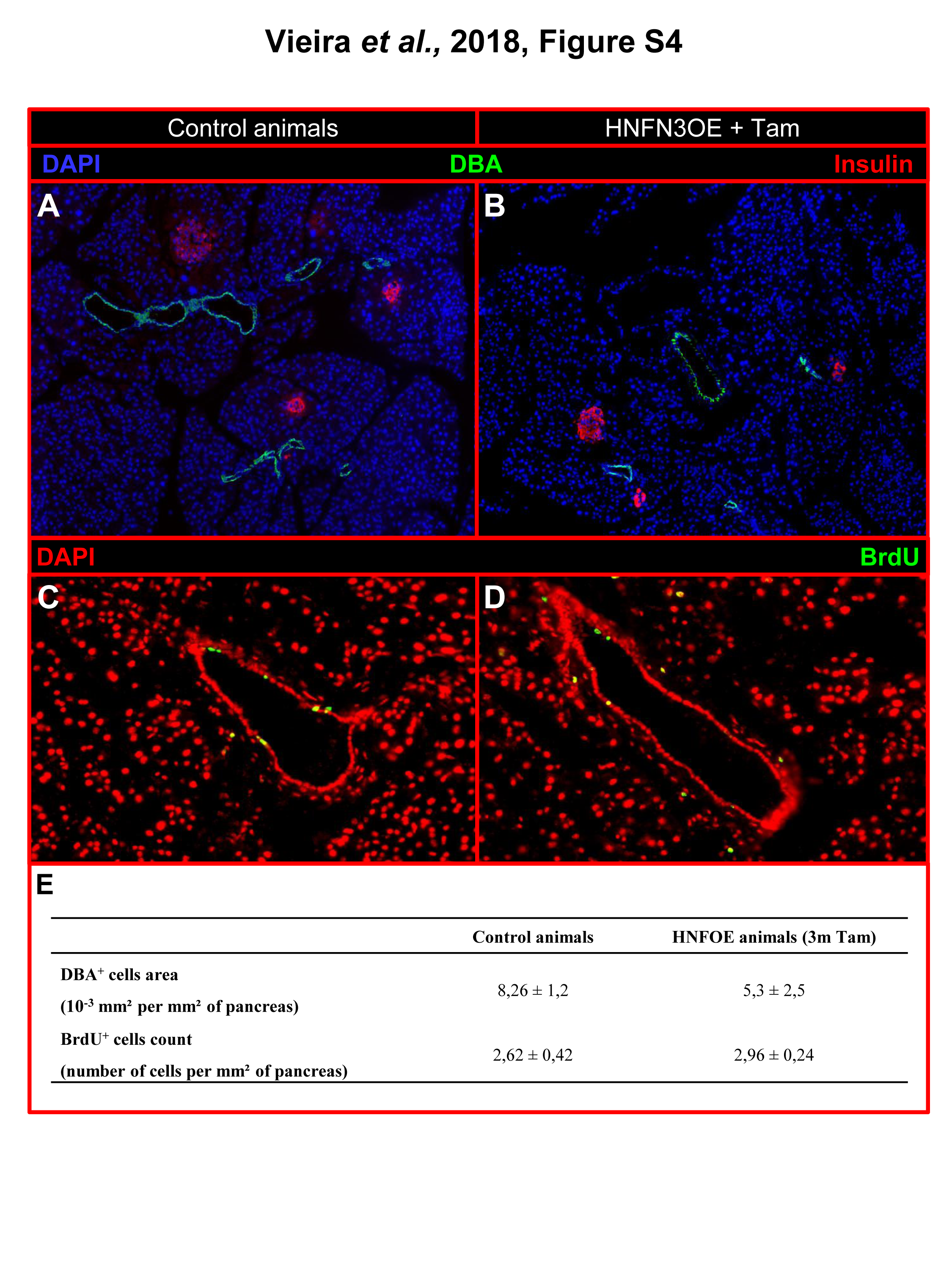

Supplement: S4 Fig — Using quantitative immunohistochemical analyses comparing ductal cells in HNFN3OE pancreata treated with vehicle (A) or Tam (B) for 12 months, no difference was detected in the number of ductal cells. Similarly, using long-term BrdU labelling (10 days prior to sacrifice), the numbers of proliferating ductal cells were found unchanged comparing vehicle- (C) and Tam-(D) treated animals (no significant difference was noted counting the numbers of BrdU+ or DBA+ ductal cells in both conditions). Ductal epithelium surface and proliferation were assessed comparing untreated animals and HNFOE Tam-treated for 3 months (E), with no significant difference observed. Statistics were performed using the Mann-Whitney test or unpaired t-test with Welch’s correction. (TIF) [file pone.0201536.s005.tif]
